# Functionalized rare earth-doped nanoparticles for breast cancer nanodiagnostic using fluorescence and CT imaging

**DOI:** 10.1186/s12951-018-0359-9

**Published:** 2018-03-22

**Authors:** Akhil Jain, Pierrick G. J. Fournier, Vladimir Mendoza-Lavaniegos, Prakhar Sengar, Fernando M. Guerra-Olvera, Enrique Iñiguez, Thomas G. Kretzschmar, Gustavo A. Hirata, Patricia Juárez

**Affiliations:** 1 Biomedical Innovation Department, Centro de Investigación Científica y de Educación Superior de Ensenada (CICESE), Carretera Ensenada-Tijuana No. 3918, Zona Playitas, C.P. 22860 Ensenada, B.C. Mexico; 2 Posgrado en Física de Materiales, Centro de Investigación Científica y de Educación Superior de Ensenada (CICESE), Carretera Ensenada-Tijuana No. 3918, Zona Playitas, C.P. 22860 Ensenada, B.C. Mexico; 3 Universidad Nacional Autónoma de México (UNAM)-Centro de Nanociencias y Nanotecnología (CNyN), Km. 107 Carretera Tijuana-Ensenada, C.P. 22860 Ensenada, B.C. Mexico; 4 Departamento de Geología, Centro de Investigación Científica y de Educación Superior de Ensenada (CICESE), Carretera Transpeninsular Ensenada-Tijuana #318, Zona Playitas, C.P. 22860 Ensenada, B.C. Mexico; 5 Centro Mexicano de Innovación en Energía Geotérmica (CeMIGeo), Rinconada del Pedregal 95, Pedregal Playitas, 22860 Ensenada, Baja California Mexico

**Keywords:** Breast cancer, Cancer detection, Luminescent nanoparticles, Folate receptor

## Abstract

**Background:**

Breast cancer is the second leading cause of cancer death among women and represents 14% of death in women around the world. The standard diagnosis method for breast tumor is mammography, which is often related with false-negative results leading to therapeutic delays and contributing indirectly to the development of metastasis. Therefore, the development of new tools that can detect breast cancer is an urgent need to reduce mortality in women. Here, we have developed Gd_2_O_3_:Eu^3+^ nanoparticles functionalized with folic acid (FA), for breast cancer detection.

**Results:**

Gd_2_O_3_:Eu^3+^ nanoparticles were synthesized by sucrose assisted combustion synthesis and functionalized with FA using EDC-NHS coupling. The FA-conjugated Gd_2_O_3_:Eu^3+^ nanoparticles exhibit strong red emission at 613 nm with a quantum yield of ~ 35%. In vitro cytotoxicity studies demonstrated that the nanoparticles had a negligible cytotoxic effect on normal 293T and T-47D breast cancer cells. Cellular uptake analysis showed significantly higher internalization of FA-conjugated RE nanoparticles into T-47D cells (*Folr*^*hi*^) compared to MDA-MB-231 breast cancer cells (*Folr*^*lo*^). In vivo confocal and CT imaging studies indicated that FA-conjugated Gd_2_O_3_:Eu^3+^ nanoparticles accumulated more efficiently in T-47D tumor xenograft compared to the MDA-MB-231 tumor. Moreover, we found that FA-conjugated Gd_2_O_3_:Eu^3+^ nanoparticles were well tolerated at high doses (300 mg/kg) in CD1 mice after an intravenous injection. Thus, FA-conjugated Gd_2_O_3_:Eu^3+^ nanoparticles have great potential to detect breast cancer.

**Conclusions:**

Our findings provide significant evidence that could permit the future clinical application of FA-conjugated Gd_2_O_3_:Eu^3+^ nanoparticles alone or in combination with the current detection methods to increase its sensitivity and precision.

**Electronic supplementary material:**

The online version of this article (10.1186/s12951-018-0359-9) contains supplementary material, which is available to authorized users.

## Background

Nanoparticles have emerged as potential tools for the diagnosis and treatment of cancer due to their selective accumulation in cancer tissue via enhanced permeation and retention (EPR) effect [1]. Due to the potential benefits of nanoparticles in cancer, various multimodality nanoparticle platforms have been developed for cancer imaging using computed tomography (CT), positron emission tomography (PET), and single-photon emission computed tomography (SPECT) [2–4]. However, due to the lack of spatial resolution and low sensitivity, most of these imaging techniques fail to detect cancer at an early stage [5, 6]. Therefore, a more sensitive dual imaging probe is needed for early cancer diagnosis that can complement clinically approved modality such as CT. On the other hand, fluorescent imaging is emerging as a powerful method to detect cancer at an early stage of the disease due to its high resolution (0.5–3 µm) and sensitivity [7, 8]. Besides fluorescence imaging is a versatile tool for biomedical imaging, therefore there is an increased demand for luminescent materials. Various luminescent nanoparticles have been developed for cancer imaging including quantum dots (QD) and inorganic nanoparticles functionalized with organic dyes [9, 10]. However, toxicity issues associated to QD and photobleaching of organic dyes limit their clinical applications [11, 12]. In contrast, rare-earth (RE) doped nanoparticles are of interest for in vivo imaging due to various advantages such as low cytotoxicity, high quantum yield, longer lifetime, narrow emission lines, large Stokes shifts, photo-stability and high chemical stability [13, 14]. Therefore, RE-doped luminescent nanoparticles have great potential as a biomarker tool for biomedical purposes.

Synthesis of various Eu^3+^:RE_2_O_3_ luminescent nanomaterials using Y, La or Gd as RE element has been reported [15–17]. Among them nanoparticles with Gd_2_O_3_ are of great interest due to its low phonon energy [18], proton relaxation [19] and scintillation [20] properties making it an excellent candidate for fluorescence imaging, MRI and X-ray CT, respectively. These Eu^3+^ doped Gd_2_O_3_ nanoparticles with different sizes and morphologies have been utilized in a variety of applications such as in optical displays, solar cells and in vivo imaging [21–23].

Physicochemical properties such as size and shape of the nanoparticles are very important for passive targeting of tumor vasculature through EPR effect. Studies elucidating the behavior of nanoparticle size, shape and surface charge on bio-distribution and biocompatibility in vivo are present in the literature [24, 25]. There are now various evidence that specificity and efficiency of passively targeted nanoparticle can be enhanced by surface modification with a specific targeting ligand [26, 27]. Therefore, for in vivo application introduction of surface modification is preferable for improving the dispersion properties and targeting potential [28].

Folate receptor alpha (FR) is highly expressed in some forms of cancers such as ovarian cancer and in up to 80% of breast cancer tumors [29]. Therefore, the addition of folic acid molecule has been used to increase the specificity of nanoparticles or drugs for cancer cells. The strong affinity of FR for its ligand folate, permit the internalization via receptor-mediated endocytosis and specific uptake FA-functionalized nanoparticles [30–32]. Hence, folic acid represents an important ligand that could be used clinically for specific targeting of breast cancer. A variety of RE-doped nanoparticles has been proposed for targeted cancer cell imaging. For instance, Setua et al. [33] reported higher cellular uptake of FA conjugated fluorescent magnetic RE nanocrystals on FR positive human nasopharyngeal carcinoma cells (KB) compared to FR depressed KB and FR negative lung cancer cells A549 control cells. Stefanakis and Ghanotakis [34] demonstrated specific targeting of HeLa cells using Tb_2_(OH)_5_NO_3_-FA nanoparticles doped with Europium.

In this study, Gd_2_O_3_:Eu^3+^ nanoparticles were produced using sucrose combustion synthesis. Gd_2_O_3_:Eu^3+^ nanoparticles (N1-Bare) were then coated with amino-silane coupling agent APTMS to introduce amine groups (N2-APTMS). Finally, these amine groups were conjugated to the carboxyl groups of FA molecule using EDC-NHS coupling mechanism to produce FA-functionalized Gd_2_O_3_:Eu^3+^ nanoparticles (N3-FA). We examined the biocompatibility and potential of folic acid-functionalized Gd_2_O_3_:Eu^3+^ nanoparticles to target breast cancer cells in vitro and in vivo using a xenograft model by dual-modal fluorescence and CT imaging. The targeting ability and toxicity of these folic acid-functionalized Gd_2_O_3_:Eu^3+^ nanoparticles is compared with N1-Bare and/or N2-APTMS. Our findings suggest that folic acid-functionalized Gd_2_O_3_:Eu^3+^ nanoparticles are promising candidates for the detection of breast cancer.

## Methods

### Materials

Gadolinium nitrate (Gd (NO_3_)_2_·6H_2_O, 99.9%) and europium nitrate (Eu (NO_3_)_2_·6H_2_O, 99.9%) were purchased from Aldrich and Alfa Aesar, respectively. Sucrose (C_12_H_22_O_11_, 99.5%), 3-aminopropyltrimethoxysilane (APTMS, 97%), toluene (ACS grade, ≥ 99.5%), folic acid (≥ 97%), *N*-(2-dimethylaminopropyl)-*N*-ethylcarbodiimide hydrochloride (EDC), *N*-hydroxysuccinimide (NHS, 98%), dimethyl sulfoxide (DMSO, ≥ 99.5%), KBr (FTIR grade, 99%) were purchased from Sigma-Aldrich.

### Synthesis of Gd_2_O_3_:Eu^3+^ nanoparticles (Gd/Eu = 0.95/0.05) (N1-Bare)

Gd_2_O_3_:Eu^3+^ nanoparticles were synthesized by sucrose combustion synthesis as previously reported [35]. Stoichiometric amounts of the metal precursors and fuels were weighed and mixed with 30 ml of distilled water under magnetic stirring for 25 min at room temperature. The obtained transparent solution was transferred to a preheated muffle furnace maintained at 380 °C. The solution was kept inside the furnace for 25 min for the complete decomposition of fuel. The synthesis was completed with the ignition of the fuel. The obtained highly porous black powder was gently crushed with a pestle and mortar. Finally, the powder was annealed at 1000 °C for 3 h to obtain a white nanocrystalline Gd_2_O_3_:Eu^3+^ powder.

### Synthesis of Gd_2_O_3_:Eu^3+^@ APTMS nanoparticles (N2-APTMS)

Freshly prepared Gd_2_O_3_:Eu^3+^ nanoparticles were dispersed in 80 ml of toluene with the help of probe sonication. After 30 min, APTMS was introduced in an equimolar ratio with Gd_2_O_3_:Eu^3+^ nanoparticles and placed under magnetic stirring, during 20 h, for efficient grafting of silane layer. The temperature of the reaction was then increased to 80 °C for 4 h for the formation of solid bonds between nanoparticle surface and silane groups. The nanoparticles were washed 4 times with ethanol and centrifuged at 6000 rpm for 15 min and dried at 65 °C overnight.

### Synthesis of folic acid-functionalized Gd_2_O_3_:Eu^3+^ nanoparticles (N3-FA)

For the surface functionalization with FA, 0.05 M folic acid was prepared in DMSO under magnetic stirring. For the activation of carboxyl groups present in FA molecule, freshly prepared 1 ml EDC (75 mM) and 1 ml NHS (150 mM) in DMSO were added to 30 ml the mixture. The reaction was allowed to continue for 4 h under an N_2_ atmosphere in the dark. Then, APTMS coated Gd_2_O_3_:Eu^3+^ nanoparticles (N2-APTMS) dispersed in PBS (pH 7.4) were introduced into the activated folic acid solution. The reaction was stirred for another 24 h under similar condition. Lastly, the nanoparticles were washed several times with DI water and ethanol and centrifuged at 6000 rpm for 15 min and dried at 65 °C overnight.

### Characterization

#### Crystalline structure

The Crystal phase of Gd_2_O_3_:Eu^3+^ nanoparticles was characterized using a Philips X’pert X-ray diffractometer with a Cu Kα radiation (λ = 0.15406 nm), scanned over a 2θ range of 20–80°.

#### Size and morphology

Transmission electron microscopy (TEM) images were acquired using JEOL-JEM-2010 operated at 200 kV. The size of nanoparticles was defined by measuring the diameters of 240 different nanoparticles. The samples were prepared by dispersing the nanoparticles in an ultrasonic bath for 15 min and then placing a few drops of the sample on 400 mesh carbon-coated copper grids.

#### Surface analysis

X-ray photoelectron spectroscopy (XPS) spectra of different Gd_2_O_3_:Eu^3+^ nanoparticle system (N1-bare, N2-APTMS, and N3-FA) was obtained by a SPECS system equipped with a PHOIBOS WAL analyzer using AlK_α_ radiation (hυ = 1486.6 eV). The scale of the spectrometer was calibrated with the reference binding energy of Ag 3d_5/2_. Fourier transform infrared spectroscopy (FTIR) spectra were recorded using a Nicolet 6700, Thermo Scientific infrared spectroscope.

#### Thermal analysis

Thermogravimetry analysis (TGA) was carried out on a TA Q600, TA Instruments under Nitrogen atmosphere, with a heating rate of 10 °C/min.

### Hydrodynamic diameter and zeta potential (ξ)

Particle size distribution and zeta potential measurements were carried out using Horiba Scientific, nanoparticle analyzer SZ-100. An aqueous suspension solution of the nanoparticles (0.25 mg/ml) was prepared in PBS (pH 7.4) by sonication in a water bath for 5 min. All the measurements were carried out at an equilibrium temperature of 25 °C and were repeated three times.

### Photoluminescence spectroscopy, quantum yield and decay time

Photoluminescence (PL) spectra and fluorescence lifetime were recorded with Hitachi F-7000 fluorescence spectrophotometer at 254 nm excitation wavelength. This excitation wavelength was chosen as it produced highest PL emission intensity. For the decay time measurements, the chopping speed of 40 Hz was used on the excitation side. The curves were fitted to a double-exponential function shown in Eq. 1.

1 $$I = I_{1 } \exp \left( { - \frac{t}{\tau 1}} \right) + I_{2} { \exp }\left( { - \frac{t}{t2}} \right)$$

where τ_1_ and τ_2_ are the decay lifetimes, and I_1_ and I_2_ are the weighing parameter obtained from the data fitting. For the double-exponential decay, the average fluorescence lifetime (τ_av_) was calculated using the following equation:

2 $$\tau_{av} = \mathop \sum \limits_{i = 1}^{N} \frac{{\alpha_{i}\uptau_{i}^{2} }}{{\alpha_{i}\uptau_{i} }}$$

PL quantum yield (QY) of Gd_2_O_3_:Eu^3+^ nanoparticles dispersed in aqueous solution was determined by using Rhodamine 6G as a reference standard (QY in ethanol = 95%), as previously reported [36]. A standard curve of PL intensity vs. absorbance (at different concentrations) was generated using multiple standards (OD values between 0.1 and 1) of Rhodamine 6G and Gd_2_O_3_:Eu^3+^ nanoparticles. The value of QY was calculated with the following formula:

$${\text{Q }} = {\text{ Q}}_{\text{R}} \left[ {{\text{m}}/{\text{m}}_{\text{R}} } \right] \, \left[ {{\text{n}}^{2} /{\text{n}}^{2}_{\text{R}} } \right]$$

where Q_R_ = known quantum yield of Rhodamine 6G; m and m_R_ = Slope of the PL intensity vs. absorption curve of the sample and the reference standard, respectively; n and n_R_ = refractive index of the solvents in which reference and the sample was dispersed: ethanol is 1.359 and PBS is 1.333 at room temperature. All the measurements were performed using a 10 mm rectangular quartz cell (Starna Cells Inc.) and repeated at least three times.

### Cell culture

Human cancer cell lines MDA-MB-231, T-47D, 293T, and PC-3 cells were obtained from American Type Culture Collection (ATCC, USA). MDA-MB-231, MCF-7, and 293T cells were maintained in DMEM (Cellgro), and T-47D and PC-3 cells were cultivated in RPMI-1640, medium (Cellgro), all supplemented with 10% FBS (Biowest), penicillin, streptomycin and amphotericin B (Cellgro). Cells were maintained at 37 °C in an incubator with humidified atmosphere, containing 5% CO_2_.

### In vitro cytotoxicity assay

The cytotoxicity of Gd_2_O_3_:Eu^3+^ nanoparticles was evaluated using a colorimetric 3-(4,5-dimethylthiazol-2-yl)-2,5-diphenyltetrazolium bromide (MTT) assay. Human embryonic kidney cells 293 T and breast cancer cell T-47D were seeded into a 96-well plate at a density of 10^5^ cells and cultured for 24 h. The culture medium was replaced with fresh medium containing various concentrations of unmodified (N1-Bare), APTMS coated (N2-APTMS) and folic acid-conjugated (N3-FA) Gd_2_O_3_:Eu^3+^ nanoparticles (6.25–100 µg/ml). After incubation for 24 h or 48, 20 µl of MTT solution (5 mg/ml in PBS) was added and incubated for 5 h. To stop the reaction and dissolve the MTT-formazan product, 100 µl of a stop buffer (0.01 M HCl containing 10% SDS) was added, and the plates were incubated for 20 h. The absorbance at 570 nm was measured using an Epoch microplate reader (Biotek). The cell viability as a percentage of the untreated control cells was calculated using the following formula:

$$\left( {{\text{Mean absorbance value of the treated group }}/{\text{ Mean absorbance value of control}}} \right) \, \times { 1}00$$

### Gene expression analysis: quantitative real-time PCR

F*olr1* mRNA level was determined using a two-step quantitative reverse transcriptase- real-time PCR (RT-qPCR). MDA-MB-231, MCF-7, T-47D, 293 T and PC-3 cells were seeded in a 12-well plate at a density of 10^4^ cells per well. Total RNA was extracted using the Gen-Elute total RNA extraction kit (Sigma-Aldrich) and reverse transcribed using SuperScript II Reverse Transcriptase (Thermo Scientific). Finally, the qPCR reactions were performed in triplicates using 10 ng of cDNA. The relative expression of F*olr1* mRNA in different human cancer cell lines was quantified by comparing it with the standard curve obtained by qPCR of cDNA pool of different cells. Gene expression of the target gene was normalized using housekeeping gene ribosomal protein L32 (*RPL32*). The primers were purchased from T4Oligo and were designed using Primer3Plus. The primer sequence used were: *Folr1* (forward, GCATTTCATCCAGGACACCT; reverse, GGTGTAGGAGGTGCGACAAT) and *RPL 32* (forward, CAGGGTTCGTAGAAGATTCAAGGG; reverse, CTTGGAGGAAACATTGTGAGCGATC).

### In-vitro fluorescence imaging and cellular uptake analysis

Cellular uptake of N3-FA in cancer cells was determined by using a confocal laser-scanning microscope (CLSM). MDA-MB-231 and T-47D cells were cultured in 35 mm glass bottom dish at a density of 2.5 × 10^5^ cells per plate and cultured for 24 h. The cells were then incubated with culture medium containing N3-FA at a concentration of 25 µg/ml for 2, 4, 6, or 8 h. Then, the cells were washed twice with PBS to remove unbound nanoparticles. Subsequently, the cells were fixed with 4% paraformaldehyde at room temperature for 5 min. The cells were visualized using an Olympus FluoView—FV 1000 confocal microscope at 60× objective. Cellular uptake of N3-FA nanoparticles was quantified by measuring fluorescent intensity of at least 90 different cells using ImageJ. The fluorescent intensity values were normalized to the total number of cells per field and expressed as corrected total cell fluorescence (CTCF) obtained by applying the following formula:

$$\begin{aligned} {\text{CTCF}} & = {\text{Integrated density}} \\ & \quad {-}\left( {{\text{Area of selected cell }} \times {\text{ Mean fluorescence of background signal}}} \right) \\ \end{aligned}$$

### Animal experiments

All animal experiments were performed in compliance with the local ethics committee. Male CD1 mice (8-week old) were obtained from Harlan-Envigo and female athymic mice (Crl: NU/NU-nuBR, 6–8 week old) were obtained from the *Unidad de Producciòn y Experimentación de Animales Laboratorio de la Universidad Autónoma Metropolitana* (Campus Xochimilco). Mice were maintained in an Optimice cage system (Animal care Systems), in a controlled environment room (temperature 24 °C and 12 h light/dark cycle). Mice received water and food (2018 Teklad Global 18% protein rodent diet) ad libitum. Mice were acclimated for at least a week before starting the experiments.

### Acute toxicity of FA-conjugated Gd_2_O_3_:Eu^3+^ nanoparticles (N3-FA) in vivo

CD1 mice were divided into 6 groups (n = 5 per group) and received intravenous tail vein injection of PBS or a solution of N3-FA in PBS (35, 50, 100, 200, and 300 mg/kg). After N3-FA administration, body weight, food intake, water intake and behavior patterns were registered daily. Mice were euthanized 7 days after administration of N3-FA in a CO_2_ chamber, and cervical dislocation was used as a secondary method of euthanasia.

### Pharmacokinetic studies

#### Luminescence of FA-conjugated Gd_2_O_3_:Eu^3+^ nanoparticle in blood

For the pharmacokinetics study, CD1 mice were divided into seven groups (n = 3 per group). Mice were administered with N3-FA nanoparticles at a dose of 200 mg/kg or with PBS. At different time interval up to 24 h, mice were anesthetized by intraperitoneal injection of 5-pentabarbitol and blood was collected using cardiac puncture. Later, the whole blood was diluted in PBS and the presence of FA-conjugated Gd_2_O_3_:Eu^3+^ nanoparticles was determined by monitoring the PL spectra using a Hitachi F-7000 fluorescent spectrophotometer.

#### Biodistribution analysis using confocal microscopy

To detect FA-conjugated Gd_2_O_3_:Eu^3+^ nanoparticles in vivo, selected organs of CD1 mice administered with N3-FA at 200 mg/kg were extracted immediately after the collection of blood samples (2 h post-injection). For biodistribution analysis, tissue sections of 6 μm were prepared using a cryostat and fixed with 4% paraformaldehyde for 10 min. Nuclei were stained using 4,6-diamidino-2-phenylindole (DAPI, 10 µg/ml) for 5 min. Later, the tissue sections were analyzed to determine the biodistribution of N3-FA using CLSM.

#### Biodistribution analysis of nanoparticles in tumor-bearing mice

Breast cancer tumor xenografts were established by subcutaneous injection of either T-47D cells (5 × 10^6^, n = 9) or MDA-MB-231 cells (2 × 10^6^, n = 3) to each flank of female nude mice (female, 6–8 weeks old). All the mice were fed autoclaved food and water. For the mice receiving T-47D cells, in addition to normal food, they were fed with 2 g/kg of chocolate spread containing 5 µl of β-estradiol (10 nM) every day. Tumor growth was monitored using a vernier caliper, and their volume was calculated using the following formula: Volume = [length × (width)^2^]/2. Three and 7 weeks after the inoculation of MDA-MB-231 and T-47D cells, respectively, mice received an intravenous inoculation of PBS, APTMS coated (N2-APTMS, 200 mg/kg) and FA-conjugated (N3-FA, 200 mg/kg) Gd_2_O_3_:Eu^3+^ nanoparticles (n = 3 per group). Organ extraction was performed 2 h after i.v. of nanoparticles and were prepared for cryosectioning. Biodistribution was analyzed using CLSM as described before and by CT imaging.

### µCT and image processing

The scanning of the tumors was done with a SkyScan 2211 nano-CT (Bruker micro-CT, Belgium). The samples were scanned using voltage of 40–50 kV, target current of 70 µA (0.1 μm Tungsten source), and exposure time of 300–350 ms, with resolution from 2.2 to 3.0 µm, depending on sample geometry, resulting in 1536 × 1920 pixels on a flat panel detector, rotation step of 0.200°, frame averaging of 4, and 360° scan to minimize artifacts produced by the combination of high-density Gd_2_O_3_:Eu^3+^ nanoparticles and low-density tissue. The reconstructed slices were obtained with NRecon v1.7.1.0 and each time histograms were forced to stay within − 750 to 5000 HU (Hounsfield Units). No staining was required. To obtain the ratio of the volume of nanoparticles/volume of the tumor, image-processing scripts were created and run with the native software of the nano-CT (CT Analyser v1.17.7.1; Bruker micro-CT, 2017). The processed volume for each tumor was fixed to ~ 0.99 mm^3^ (for comparison purpose) consisting of cylindrical sections with a diameter and height of 1.5 and 0.56 mm, respectively.

### Statistical analysis

Statistical analyses were performed using GraphPad Prism v5.0 software (GraphPad Software, Inc). Comparisons of three or more groups were conducted with a 1-way ANOVA test, followed by a Bonferroni’s post-test. For responses that were affected by two variables, a 2-way ANOVA with a Bonferroni’s or Tukey post-test was used. Results are expressed as mean ± SEM and a P ≤ 0.05 was considered significant.

## Results

### Synthesis and characterization of Gd_2_O_3_:Eu^3+^ nanoparticles

**Scheme 1:**
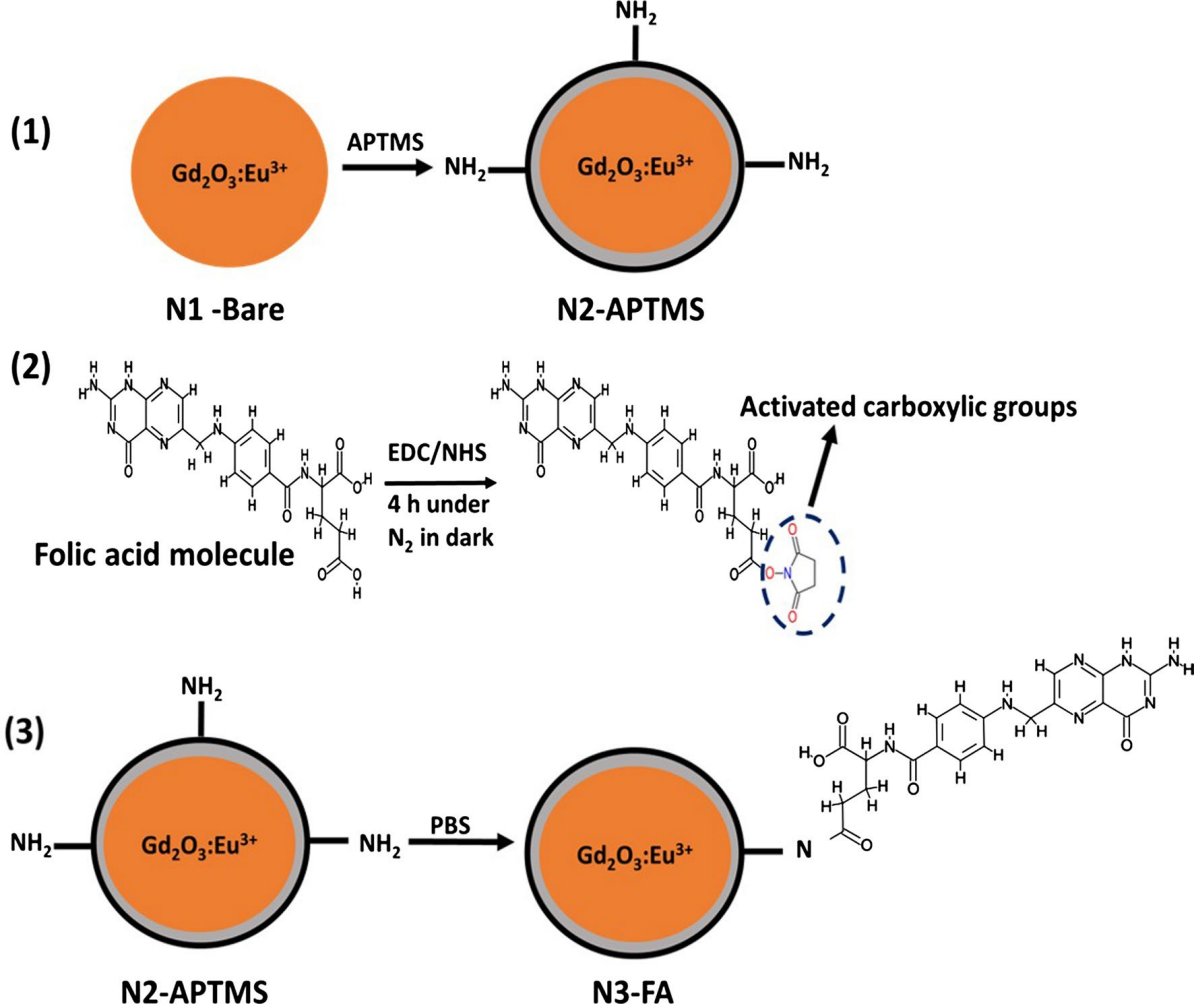
Schematic representation of synthesis procedure involved in the development of folic acid functionalized Gd_2_O_3_:Eu^3+^ nanoparticles

**Fig. 1:**
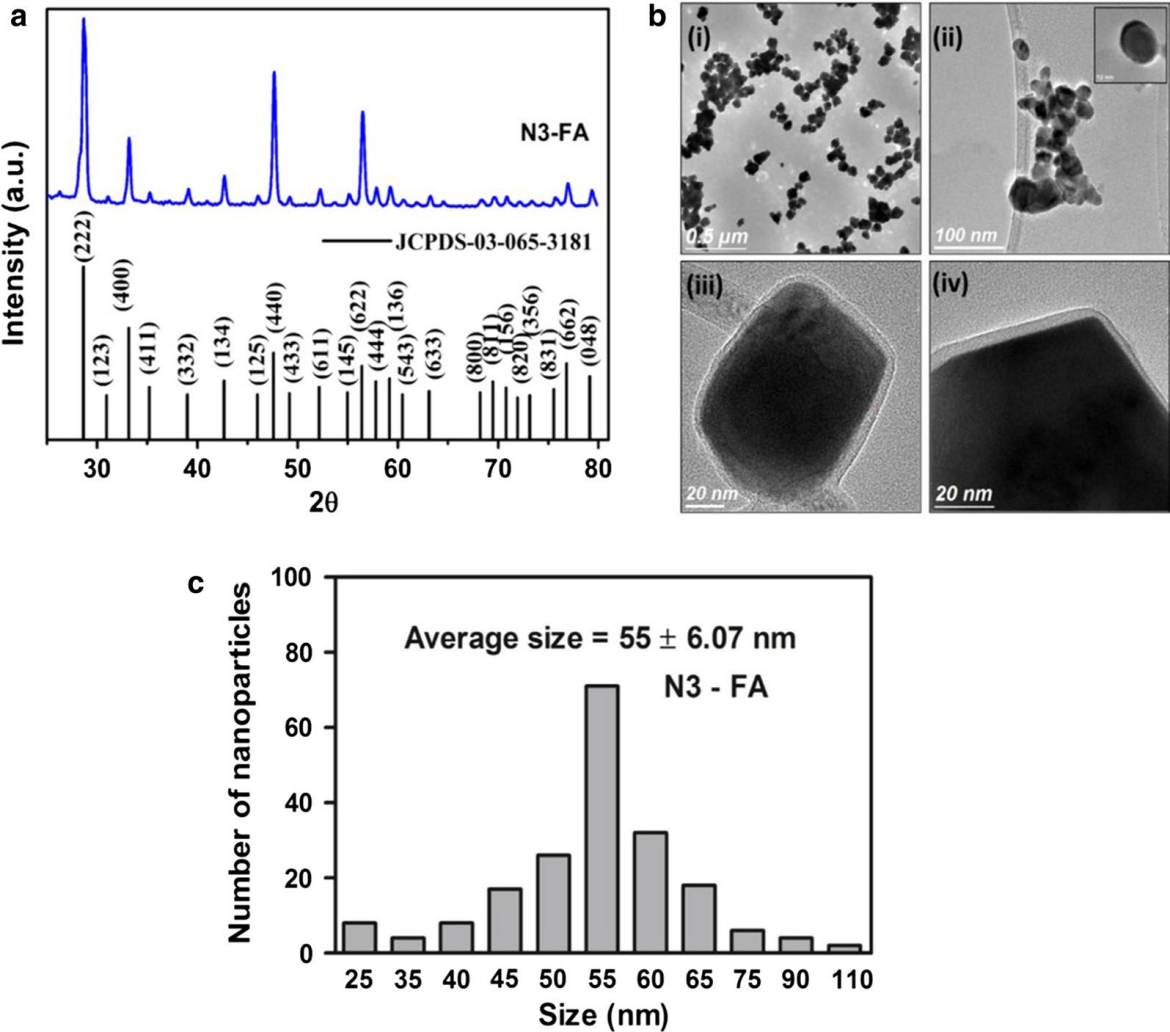
Characterization of Gd_2_O_3_:Eu^3+^ nanoparticles. **a** X-Ray diffraction pattern of N3-FA. **b** TEM images (**i** and **ii**) N1-Bare (**iii** and **iv**) N3-FA. **c** Histogram of particle size distribution of N3-FA obtained from TEM analysis

Gd_2_O_3_:Eu^3+^ nanoparticles were produced using sucrose combustion synthesis and post-annealed at 1000 °C for 3 h to improve the crystalline structure. The schematic illustration of synthesis procedure of Gd_2_O_3_:Eu^3+^@ APTMS @ FA is presented in Scheme 1. In general, combustion synthesis produces nanoparticles with good crystallinity due to high reaction temperature. XRD pattern of N3-FA was in agreement with JCPDS-03-065-3181, indicating the presence of a cubic crystal phase of Gd_2_O_3_ (Fig. 1a). The presence of an intense and sharp peak with indices (222) suggested the presence of a well crystalline structure. The average crystallite size calculated using Scherrer’s formula was 36.89 ± 0.12 nm for N3-FA. TEM analysis indicated the presence of a few large agglomerates, probably due to the presence of weak van der Walls forces between the nanoparticles and the average size of N1-Bare was 45 ± 3.6 nm (Fig. 1b(i and ii)). After APTMS coating, the silane layer was estimated to be around 3 ± 1.5 nm (Fig. 1b(iii and iv)), and the average size was further increased to 55 ± 6.07 nm for N3-FA (Fig. 1c).

**Fig. 2:**
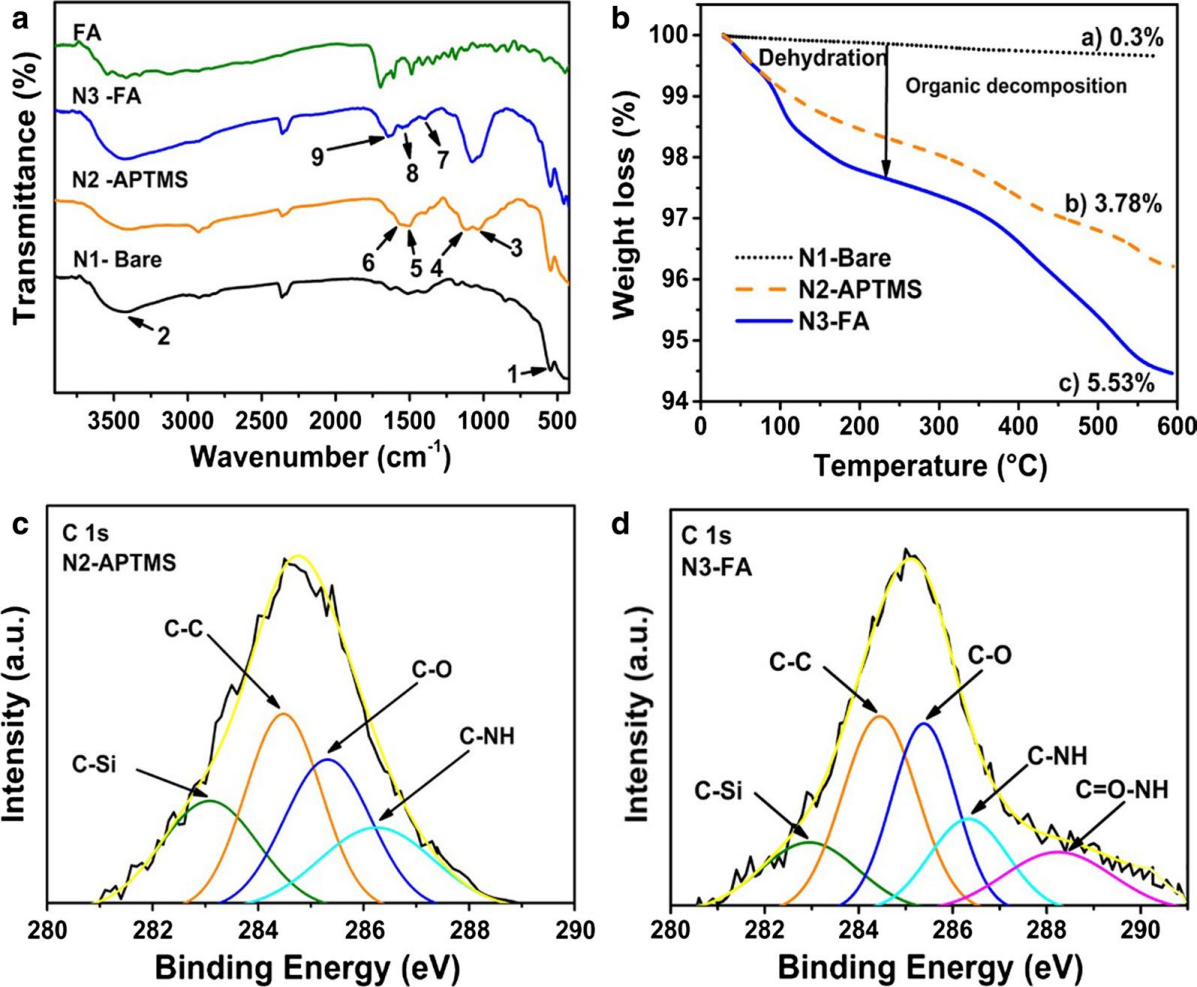
Surface characterization of Gd_2_O_3_:Eu^3+^ nanoparticles. **a** FTIR spectra, **b** TGA curves of unmodified (N1-bare), APTMS coated (N2-APTMS), and folic acid-functionalized Gd_2_O_3_:Eu^3+^ nanoparticles (N3-FA); high-resolution C 1s XPS spectra of **c** N2-APTMS and **d** N3-FA

FTIR measurements were carried out to characterize the surface functional groups present on different Gd_2_O_3_:Eu^3+^ nanoparticles (Fig. 2a). A peak at ~ 543 cm^−1^ is characteristic of the lattice vibration of Gd-O (peak 1) was detected in all the samples. A well-defined peak at 1029 cm^−1^ (peak 3) was observed in N2-APTMS due to the formation of Si–O–Si linkage. After APTMS-coating, new peaks appeared in the spectra of N2-APTMS at 1132 cm^−1^ (peak 4), 1492 cm^−1^ (peak 5) and 1567 cm^−1^ (peak 6), characteristics vibration of the Si–C groups, the symmetric –NH_3_^+^ deformation mode and of scissor vibration of the terminal –NH_2_ groups, respectively [37]. A weak absorption peak was observed at 1400 cm^−1^ (peak 7) in N3-FA, which could be attributed to the stretching vibrational mode of C=C groups present in FA molecules. The characteristic peaks at ~ 1545 and 1647 cm^−1^ (peak 8 and 9) have been characterized as the stretching vibration mode of secondary amide bonds (C=O–NH).

Figure 2b shows the TGA analysis of the different Gd_2_O_3_:Eu^3+^ nanoparticle systems. Weight loss in N2-APTMS and N3-FA at low temperature (≤ 200 °C) was due to the dehydration of water molecules attached to the surface of nanoparticles. The weight loss due to the presence of organic moieties (> 200 °C) was estimated to be 3.78 and 5.53% for N2-APTMS and N3-FA, respectively. The higher weight loss in N3-FA compared to N2-APTMS is due to the conjugation of folic acid molecules onto the surface of N2-APTMS.

Conjugation of FA to APTMS coated nanoparticles was further confirmed by XPS analysis. Preliminary confirmation of FA conjugation was obtained through the presence of an intense nitrogen peak in N3-FA compared to N2-APTMS, due to the presence of 7 nitrogen atoms in FA molecule (Additional file 1: Figure S1). Additionally, the high-resolution spectrum of C 1s of N2-APTMS and N3-FA are shown in Fig. 2c, d. Deconvolution of C 1s showed the existence of amide bond in N3-FA. The C 1s spectrum of N3-FA was well fitted into five peaks located at 282.8, 284.4, 285.2, 286.3 and 288.4 eV. The lowest binding energy (B.E.) component (282.8 eV) was associated with peaks that arise due to the presence of C–Si bonds in APTMS molecules. Higher B.E. components at 284.4, 285.2 and 286.3 eV has been assigned to C–C/C=C, C–O and C–NH_3_^+^/C–NH bonds [38], the presence of these peaks can be seen in both APTMS coated (Fig. 2c) and FA-functionalized Gd_2_O_3_:Eu^3+^ nanoparticles (Fig. 2d). In addition, another higher energy component at 288.4 eV (Fig. 2d) was obtained specifically in N3-FA, which corresponds to the backbone of amide bond (NH–C=O) [38]. The presence of peak due to amide bonding confirms the successful conjugation of NH_2_ groups of Gd_2_O_3_:Eu^3+^ @ APTMS (N2-APTMS) with –COO^−^ groups of FA.

The nanoparticle surface charge is directly influenced by the chemical composition of the coating, which also affects the stability of nanoparticles in aqueous solution. The colloidal stability of the different Gd_2_O_3_:Eu^3+^ nanoparticle system (N1-Bare, N2-APTMS, and N3-FA) was determined by measuring zeta potential in PBS (Table 1). The obtained values indicated a weak colloidal stability of Gd_2_O_3_:Eu^3+^ nanoparticles (N1-Bare) in PBS. However, the colloidal stability was improved after surface functionalization with APTMS (N2-APTMS) and folic acid (N3-FA), which could be due to the introduction of surface reactive amine groups. We further determined the hydrodynamic diameter of the nanoparticles using DLS. The hydrodynamic diameter of N1-bare was around 126 nm indicating the presence of agglomeration between the nanoparticles (Table 1). The hydrodynamic diameter of N2-APTMS and N3-FA nanoparticles was increased to 134 and 143 nm, respectively, due to the introduction of silane layer and functionalization process. The polydispersity index (PDI) values of Gd_2_O_3_:Eu^3+^ nanoparticle system was in the range of 0.27–0.31, thus indicating mid-range of particle size distribution. Overall, these results confirmed the synthesis of FA-functionalized Gd_2_O_3_:Eu^3+^ nanoparticles with optimal dispersion property.

**Table 1:** Hydrodynamic diameter (d), zeta potential (ζ) and polydispersity index of different Gd_2_O_3_:Eu^3+^ nanoparticles

Particle system	PBS (pH 7.4)		
	ζ (mV)	d (nm)	PDI
N1-Bare	+ 9.9 ± 3.22	126	0.315
N2-APTMS	+ 16.2 ± 1.05	134	0.298
N3-FA	+ 23.2 ± 1.84	143	0.279

Error bars represent the standard deviation of three individual experiments. All the measurements were performed in PBS (pH 7.4)

### Photoluminescence (PL) properties

**Fig. 3:**
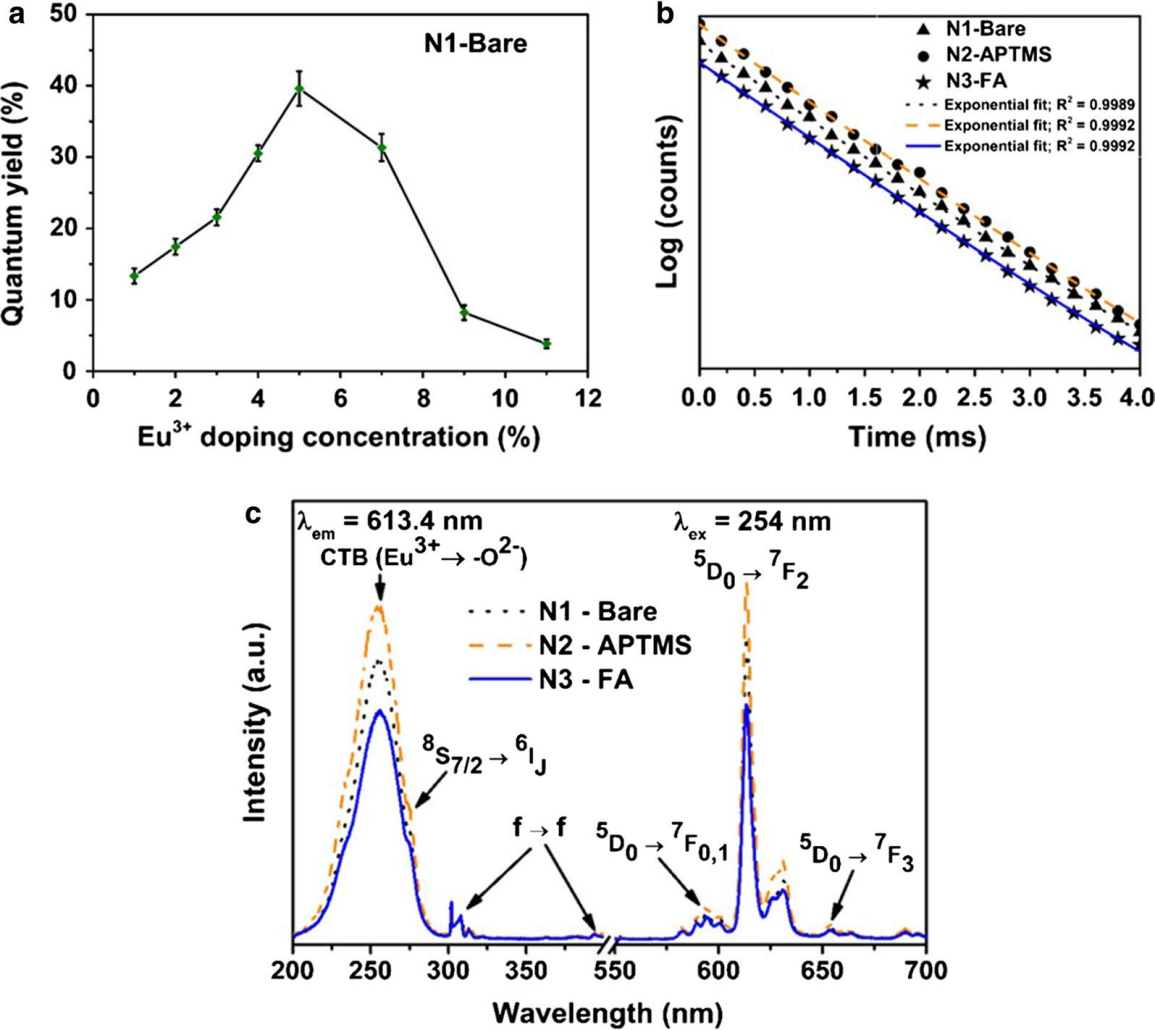
Photoluminescence properties of Gd_2_O_3_:Eu^3+^ nanoparticles. **a** Quantum yield of Gd_2_O_3_:Eu^3+^ as a function of Eu^3+^ doping concentration, **b** fluorescent decay time curves, and **c** photoluminescence excitation and emission spectra. All the measurements were performed in PBS (pH 7.4)

Rare-earth (RE) doped down-conversion luminescent nanoparticles have been extensively studied due to their unique optical properties. We have characterized the quantum yield, photoluminescence excitation/emission spectra, quantum yield, and fluorescence lifetime of the proposed Gd_2_O_3_:Eu^3+^ nanoparticle system for cancer detection and bio-imaging. Quantum yield (QY) is an essential parameter required for high contrast bio-imaging and defines the photoluminescence efficiency of a nanophosphor. It is well known that the efficiency of a radiative recombination process is directly proportional to the quantum yield. Figure 3a shows the effect of Eu^3+^ doping concentration on the QY of Gd_2_O_3_:Eu^3+^ nanoparticles. The QY of the nanoparticles reached its highest value of ~ 40% at a Eu^3+^ concentration of 5%. Above 5%, a decrease in the QY was observed, which could be attributed to luminescence concentration quenching. At these europium concentrations, the distance between the neighboring activator ions (Eu^3+^) becomes smaller causing Eu^3+^ ions to move around the host lattice causing resonant energy transfer thus sending excitation energy to the quenching center. The quantum yield of different Gd_2_O_3_:Eu^3+^ (Eu^3+^ doping concentration = 5%) nanoparticle system was determined in the aqueous colloidal form using rhodamine 6G as a reference standard (Table 2). A maximum of ~ 42% QY was obtained for N2-APTMS, which is slightly higher than N1-bare nanoparticles (~ 40%) indicating that surface coating with amino-silane increase the radiative emission from Gd_2_O_3_:Eu^3+^ nanoparticles. On the other hand, the calculated value of QY for FA-functionalized nanoparticles (N3) was ~ 35%, indicating that the folic acid functionalization causes an increase in non-radiative emission process. Furthermore, fluorescence lifetime (τ) measurements were carried out, which is an important parameter for overcoming the background auto-fluorescence [39]. The lifetime decay curves of different Gd_2_O_3_:Eu^3+^ nanoparticles system is shown in Fig. 3b. The calculated average fluorescence lifetime (τ_av_) values of the Gd_2_O_3_:Eu^3+^ nanoparticles (N1-Bare, N2-APTMS, and N3-FA) were around ~ 1 ms (Table 2).

**Table 2:** Average decay time and absolute quantum yield values of different Gd_2_O_3_:Eu^3+^ nanoparticle system measured in aqueous colloidal form

Nanoparticle system	Average decay time (ms)	QY (%)
N1-Bare	1.076 ± 0.003	39.6 ± 2.43
N2-APTMS	1.114 ± 0.002	41.4 ± 3.03
N3-FA	1.061 ± 0.006	35.3 ± 1.79

Error bars represent the standard deviation of three individual experiments

The photoluminescence excitation and emission spectra of the nanoparticle systems, measured in PBS is shown in Fig. 3c. The excitation spectra of Gd_2_O_3_:Eu^3+^ (Eu^3+^ doping concentration = 5%) nanoparticles consisted of three main excitation bands with peaks located at 254, 275 and at 394 nm. The characteristic peak at 254 nm corresponds to the charge transfer band which arise due to the transfer of an electron to 4f orbital of europium from 2p orbital of oxygen. Other peaks observed at ~ 275 and 394 nm are associated with 4f-4f electronic transitions of Gd^3+^ and Eu^3+^, respectively. The emission spectra was observed after excitation with 254 nm, which produced the highest PL intensity. The observed emission bands are the result of the transition from ^5^D_0_ excited state level of Eu^3+^ to ^7^F_J_ (J = 0, 1, 2, 3) ground state level. A group of peaks around ~ 580 nm was attributed to the transition from ^5^D_0_ → ^7^F_0_ and another peak located at 593 nm to magnetic dipole allowed by ^5^D_0_ → ^7^F_1_ level. The observed red emission of Gd_2_O_3_:Eu^3+^ nanoparticle was due to the emission peak at 613.4 nm, which is due to an electric dipole associated with ^5^D_0_ → ^7^F_2_ transition of Eu^3+^. We observed variations in the PL emission intensity of N1-Bare, N2-APTMS and N3-FA systems. The PL emission intensity of Gd_2_O_3_:Eu^3+^ nanoparticles was increased after APTMS coating, which could be attributed to the removal of hydroxyl groups (Fig. 2a, peak 2), well known for quenching radiative process from nanophosphor materials. Additionally, the decrease in reflectivity of UV light from the surface of the nanoparticles due to the presence of silane layer could be another reason for the increased PL intensity. Finally, the PL intensity of N3-FA nanoparticles was decreased, which is probably due to the introduction of folic acid that contains a large number of organic groups. The high energetic vibration of organic groups near the surface of a nanophosphor has also been shown to reduce PL intensity by increasing non-radiative recombination process [40].

### In vitro cytotoxicity

**Fig. 4:**
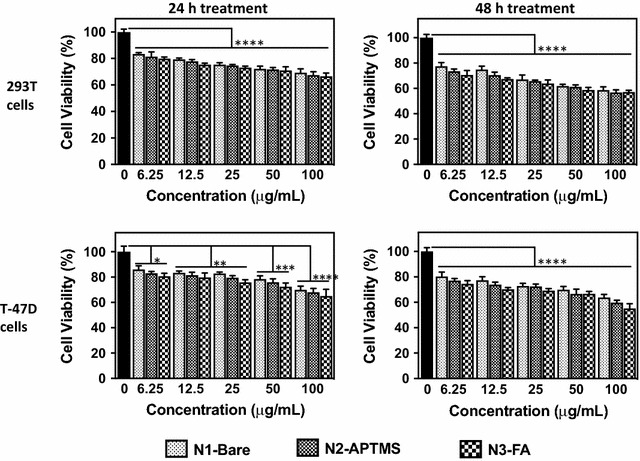
Effect of Gd_2_O_3_:Eu^3+^ nanoparticles on cell viability. Human embryonic kidney cells 293T and human breast cancer cells T-47D were cultured in increasing concentration ofunmodified (N1-bare), APTMS coated (N2-APTMS), and folic acid-functionalized Gd_2_O_3_:Eu^3+^ nanoparticles (N3-FA) for 24 or 48 h, before measuring cell viability. The experiment was run in triplicate and OD570 nm is expressed as % of control (0 µg/ml nanoparticles). Results are expressed as the mean ± SEM *P < 0.05; **P < 0.01; ***P < 0.001; and ****P < 0.0001 obtained using 2-way ANOVA with a Tukey post-test

The significant photoluminescence and physicochemical properties of Gd_2_O_3_:Eu^3+^ nanoparticles encouraged us to study their potential in cancer imaging. As a first step, we determined the cytotoxic effect of Gd_2_O_3_:Eu^3+^ nanoparticles in vitro on the human breast cancer cells T-47D and the 293T cell line derived from normal human embryonic kidney cells. T-47D and 293T cells were cultured in the presence or absence of increasing concentrations of nanoparticles (N1-Bare, N2-APTMS, or N3-FA) and cell viability was evaluated after 24 or 48 h of treatment using a colorimetric MTT assay. A continuous 24 h- and 48 h-treatment induced a significant time- and dose-dependent decrease of the viability of the two cell lines tested when compared to untreated cells, starting at a concentration as low as 6.25 µg/ml (Fig. 4). At lower concentrations, the decrease remained limited, reaching 20% after 48 h, at 6.25 µg/ml for the 293T cells. At higher concentrations, the decrease of viability was limited to 38% at 100 µg/ml of nanoparticles for T-47D, after 48 h. Interestingly, there was no significant difference when comparing the different formulations of nanoparticles (N1-Bare, N2-APTMS, and N3-FA), suggesting that the decreased viability was due to the core Gd_2_O_3_:Eu^3+^ nanoparticle itself and not the functionalization (Fig. 4).

### Folate receptor expression and cellular uptake

**Fig. 5:**
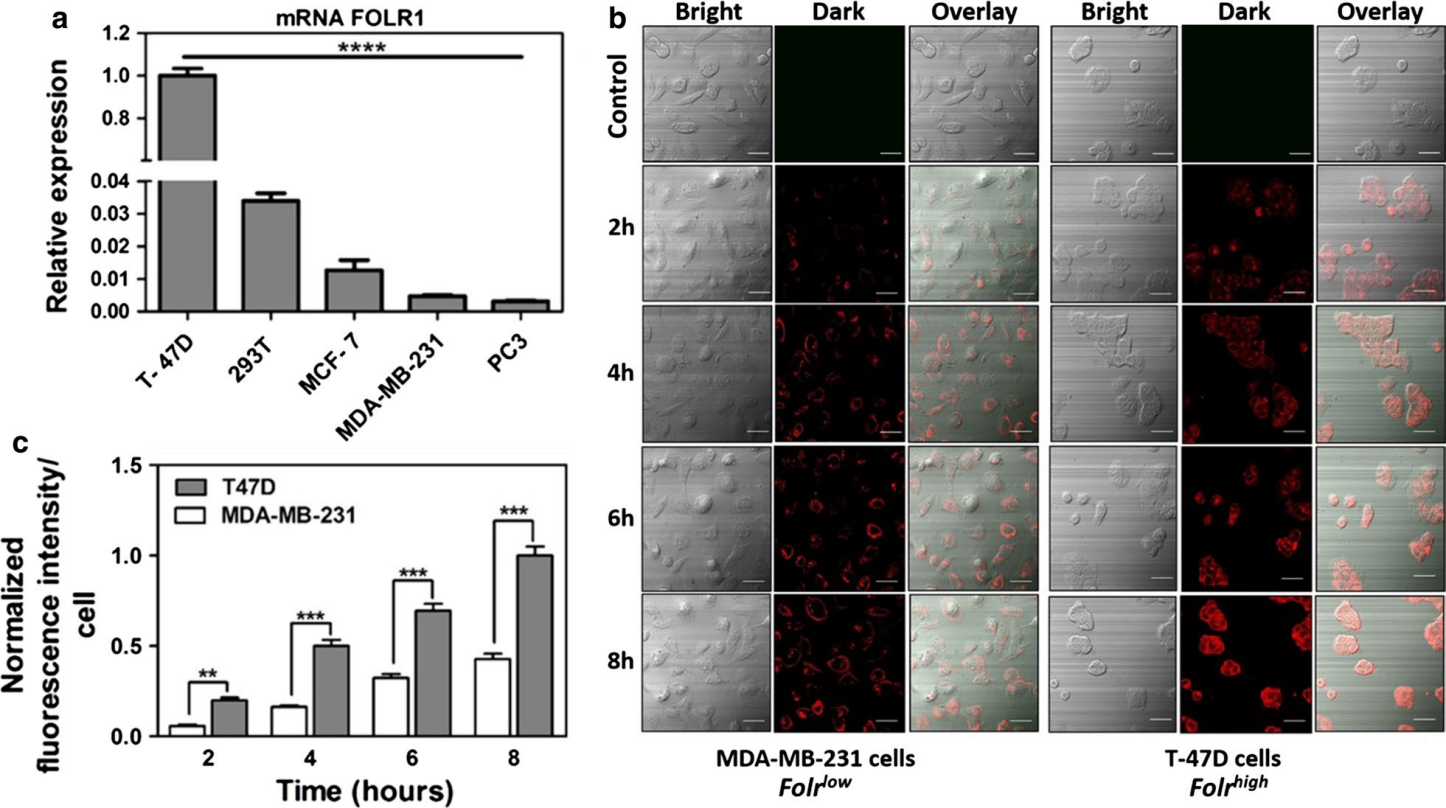
In vitro cellular uptake and receptor expression analysis. **a** Relative expression of *Folr1* mRNA in different cancer cell lines. ****P < 0.0001 vs T-47D cells using 1-way ANOVA, **b** confocal microscopy images of MDA-MB-231 and T-47D breast cancer cell lines incubated with folic acid-functionalized Gd_2_O_3_:Eu^3+^ nanoparticles (N3-FA) for different time intervals, scale bar = 20 µm, and **c** mean fluorescent intensity as a function of cellular uptake of N3-FA nanoparticles on MDA-MB-231 and T-47D cells. All the values were normalized with fluorescent intensity obtained with N3-FA treatment of T-47D cells at 8 h. Error bars represent the SEM of fluorescent intensity of at least 90 different cells. **P < 0.01; ***P < 0.001 obtained using 2-way ANOVA with a Bonferroni post-test

To increase the affinity of the Gd_2_O_3_:Eu^3+^ nanoparticles for cancer cells and detect tumors but also to decrease the risk of internalization in normal, which could lead to toxicity and side-effects, it was decided to functionalize the nanoparticles with folic acid since its receptor, the folate receptor, is often overexpressed in cancer. To reproduce this in vitro, we sought to identify a cell line with a high expression of folate receptor (*Folr1*^*hi*^), and we determined the level of expression of folate receptor *Folr1* in different human cancer cell lines using RT-qPCR. T-47D human breast cancer cells showed a significantly higher expression of *Folr1* when compared to kidney cells (293T) or other breast cancer cell lines (MCF-7 and MDA-MB-231) and prostate cancer cell line (PC-3) (Fig. 5a). To evaluate the targeting ability of FA-functionalized Gd_2_O_3_:Eu^3+^ nanoparticles (N3-FA), we examined their cellular uptake in T-47D (*Folr1*^*hi*^) and MDA-MB-231 (*Folr1*^*lo*^) human breast cancer cell lines. After 2 h incubation, the red fluorescent signal from N3-FA of T-47D cells was strong and located inside the cytoplasm, while in MDA-MB-231 cells the fluorescence was mostly found on the outer membrane, and significantly lower than in T-47D cells, even after longer incubation time (Fig. 5b, c). On the contrary, the internalization of N3-FA nanoparticles into the cytoplasm of T-47D cell lines continuously increased with incubation time suggesting that the N3-FA nanoparticles were specifically internalized into T-47D cells. This difference in N3-FA uptake between *Folr1*^*hi*^ and *Folr1*^*lo*^ cells is likely due to the affinity of Folr1 towards the folic acid molecules conjugated to the surface of Gd_2_O_3_:Eu^3+^ nanoparticles. These results indicate the feasibility of using folic acid functionalized-Gd_2_O_3_:Eu^3+^ nanoparticles as an efficient fluorescent probe for cancer cell imaging and, eventually, targeted drug delivery.

### In vivo acute toxicity and pharmacokinetics of folic acid conjugated-Gd_2_O_3_:Eu^3+^ nanoparticles (N3-FA)

**Fig. 6:**
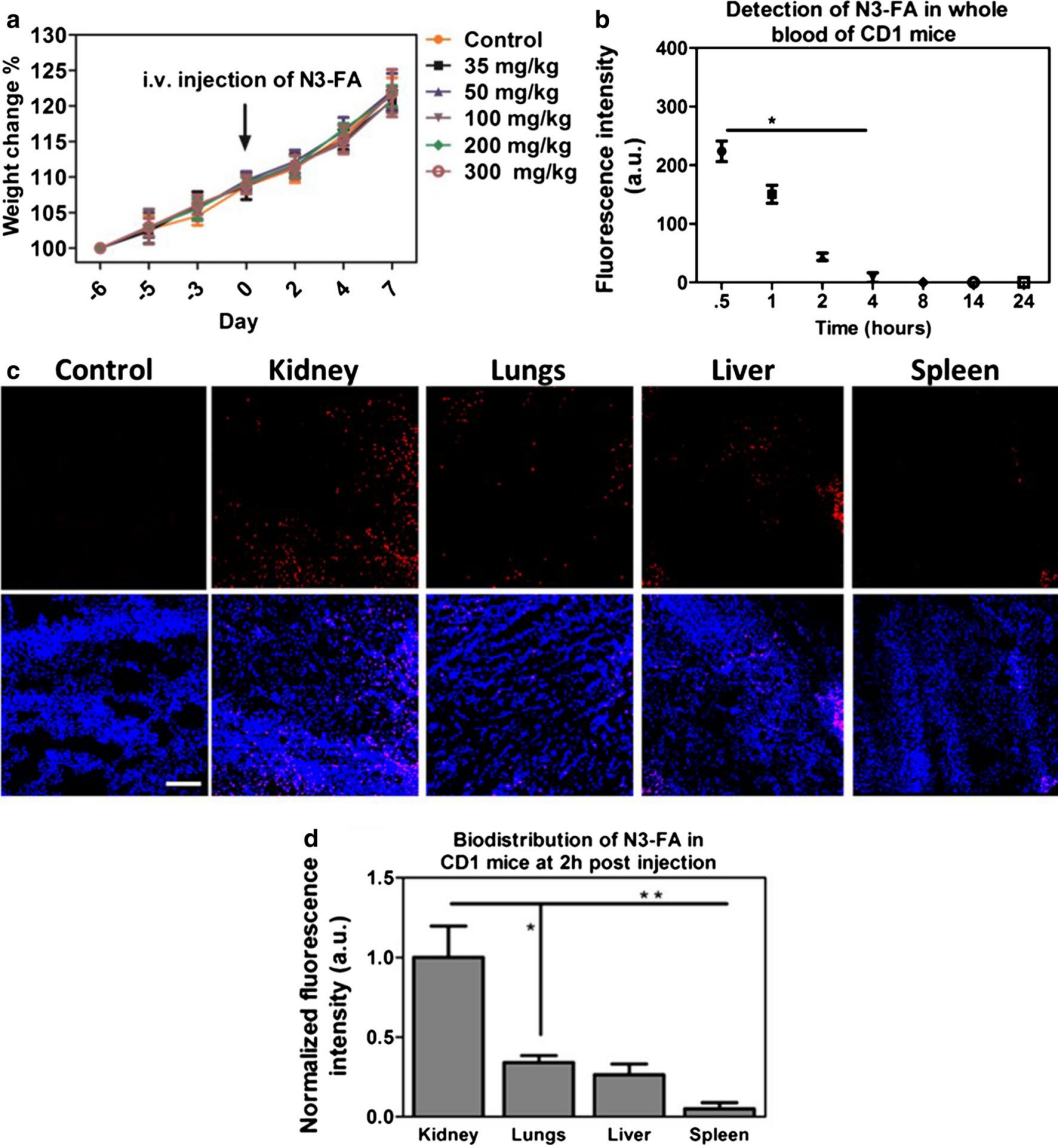
Acute toxicity and pharmacokinetic characterization of folic acid-functionalized Gd_2_O_3_:Eu^3+^ nanoparticles (N3-FA) in CD1 mice. **a** Change in body weight of CD1 mice after i.v. injection of N3-FA at different doses, **b** detection of N3-FA in whole blood of CD1 mice using fluorescent spectrophotometer at various time intervals, **c** biodistribution of N3-FA in different organs of CD1 mice 2 h after i.v. injection at a dose of 200 mg/kg, analyzed using CLSM of tissue sections, Scale bar = 50 µm, and **d** mean fluorescent intensity as a function of N3-FA accumulation in different organs of CD1 mice. *P < 0.05; **P < 0.01 vs kidney using 1-way ANOVA with a Bonferroni post-test

First, we determine the highest dose of nanoparticles that could be tolerated by the animals, N3-FA were administered intravenously (i.v.) via the tail vein with increasing doses (from 35 to 300 mg/kg) in CD1 mice. The symptoms of preliminary response to the nanoparticle injection, behavior pattern, food and water intake, and change in body weight were monitored until 7 days. The percentage of change in body weight of the mice during the course of the experiment is shown in Fig. 6a. We found that there was no significant difference in the body weight of the mice injected with N3-FA compared to control mice that received PBS. In addition, food or water intake were not different in mice that received N3-FA compared with control (Additional file 1: Figure S3) and none of the mice inoculated died regardless of N3-FA dose, suggesting that even at high dose nanoparticles were well tolerated in mice.

Next, we question for how long we can find the nanoparticles in the blood circulation. Taking into account the optimal QY of N3-FA, their presence in blood samples of CD1 mice after i.v. injection was analyzed by monitoring their emission spectra. Figure 6b shows that the nanoparticles were detected in whole blood up to 2 h after inoculation, which could be attributed to rapid clearance of N3-FA from blood circulation. Further, in vivo biodistribution of N3-FA nanoparticles was analyzed in different organs of CD1 mice, 2 h after intravenous injection. N3-FA was detected in kidneys, liver, lungs, and spleen (Fig. 6c, d). Highest levels of N3-FA were detected in kidneys, while liver and lungs showed a lower, but similar accumulation. Spleen presented very weak fluorescence signal, suggesting poor uptake of N3-FA by spleen cells. The significantly higher accumulation of N3-FA in kidney compared to other organs suggests their rapid metabolism and clearance from the body (Fig. 6d). The presence of nanoparticles in liver, lungs, and spleen has been attributed to the distribution of mononuclear phagocytes [25].

### Biodistribution and tumor-targeting ability of folic acid-conjugated Gd_2_O_3_:Eu^3+^ nanoparticles in breast cancer xenograft models analyzed using CLSM

**Fig. 7:**
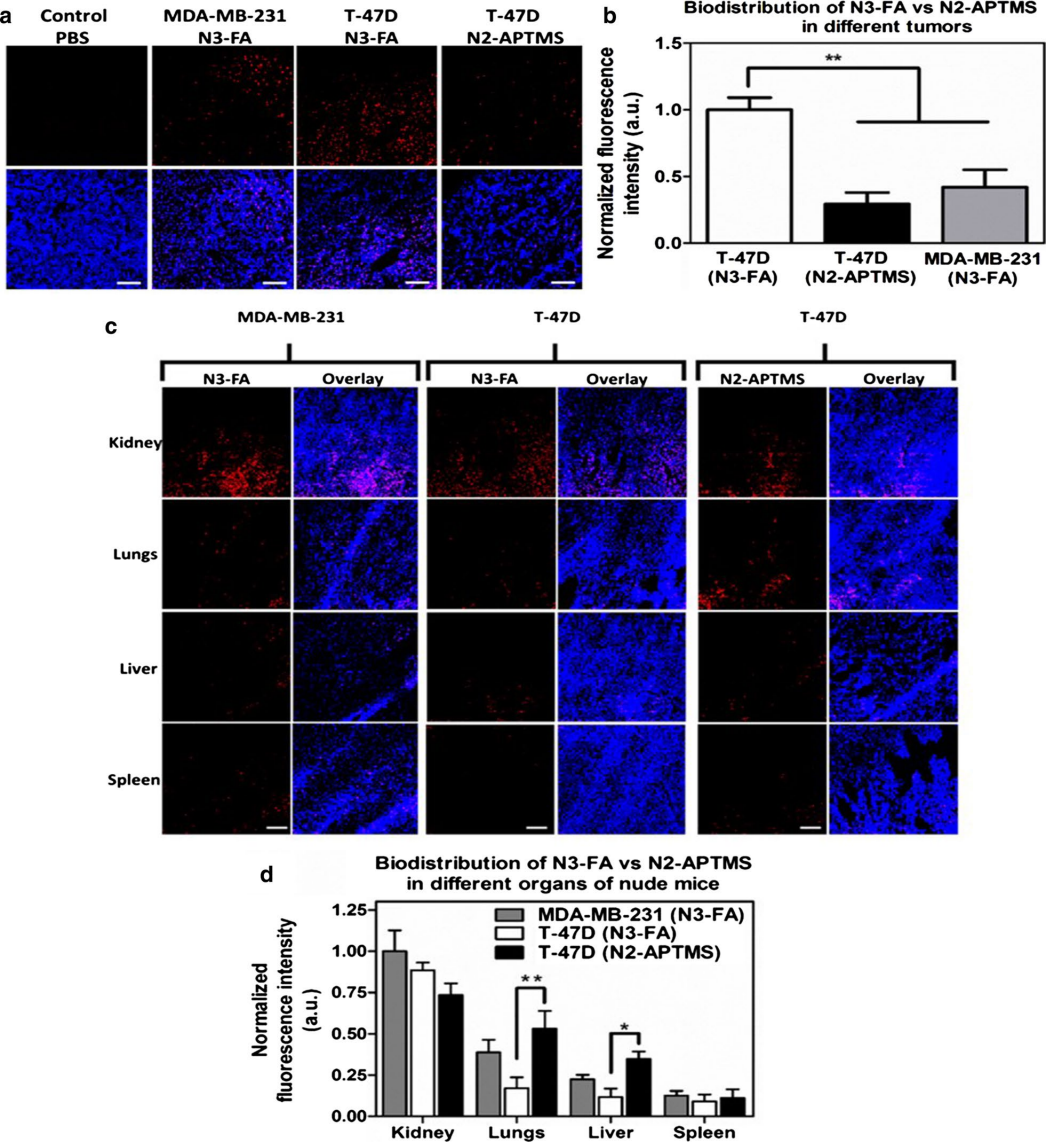
Biodistribution of APTMS conjugated (N2-APTMS), and folic acid-functionalized Gd_2_O_3_:Eu^3+^ nanoparticles (N3-FA) in breast cancer tumor xenografts. **a** Confocal microscopy images of different tumor sections showing biodistribution of N2-APTMS and N3-FA at 2 h after i.v. injection, **b** mean fluorescent intensity as a function of N3-FA accumulation in tumors of MDA-MB-231 and T-47D tumor-bearing mice. **P < 0.01 vs. T-47D tumors with N3-FA treatment using 1-way ANOVA with a Bonferroni post-test, **c** biodistribution of N2-APTMS and N3-FA in different organs of tumor-bearing mice, analyzed using confocal microscopy images 2 h after i.v. injection, **d** mean fluorescent intensity as a function of N3-FA accumulation in different organs of MDA-MB-231 and T-47D tumor-bearing mice. *P < 0.05; **P < 0.01 vs. kidney of MDA-MB-231 tumor-bearing mice treated with N3-FA using 2-way ANOVA with a Bonferroni post-test. Error bars represent the SEM of fluorescent intensity of 3 different tissues. Scale bar = 50 µm

To evaluate the targeting potential of FA-conjugated nanoparticles in tumor-bearing mice, we inoculated T-47D (*Folr1*^*hi*^) and MDA-MB-231 cells (*Folr1*^*lo*^) in immunodeficient mice. The mice bearing T-47D tumors were injected either with N3-FA (n = 3) or N2-APTMS (n = 3; control), and the mice with MDA-MB-231 tumors were injected with N3-FA (n = 3). Organs of the mice were collected 2 h post injection of nanoparticles, and their distribution was analyzed using CLSM. A strong fluorescence was detected in T-47D tumors injected with N3-FA (Fig. 7a), indicating fast tumor uptake. In contrast, N2-APTMS showed poor accumulation in tumors compared to N3-FA, this is due to the fact that N2-APTMS do not have folic acid-functionalization and their accumulation would be solely due to the EPR effect. Furthermore, N3-FA showed poor accumulation in MDA-MB-231 tumors, which could be attributed to the relatively low levels of folate receptors (Fig. 7b). These results showed that active targeting of folate receptors using N3-FA significantly increased its accumulation in T-47D tumors (*Folr1*^*hi*^) compared to the N2-APTMS. Moreover, poor uptake in MDA-MB-231 tumors (*Folr1*^*lo*^) demonstrated high specificity of FA-conjugated Gd_2_O_3_:Eu^3+^ nanoparticles (N3-FA) towards folate receptor-expressing tumors. Figure 7c shows CLSM images of different organs of tumor-bearing mice. A high fluorescent signal in kidney was observed in all mouse groups irrespective of the mice model and the type of nanoparticles injected, suggesting that Gd_2_O_3_:Eu^3+^ nanoparticles are mainly metabolized through the urinary system. In a similar way that was observed in CD1 mice, diffused distribution of N2-APTMS and N3-FA was seen in lungs, liver, and spleen. Further, quantification of fluorescent intensity (Fig. 7d) confirmed that N2-APTMS and N3-FA have similar uptake profile in kidney, while the non-specific uptake by lungs and liver is significantly reduced after folic acid functionalization. Overall, these results demonstrate the targeting potential of FA-conjugated Gd_2_O_3_:Eu^3+^ nanoparticles (N3-FA) in vivo and confirm their specificity towards *Folr1*^*hi*^ tumors such as those observed in intra-ductal carcinoma.

### Tumor accumulation of Gd_2_O_3_:Eu^3+^ nanoparticles analyzed using CT imaging

**Fig. 8:**
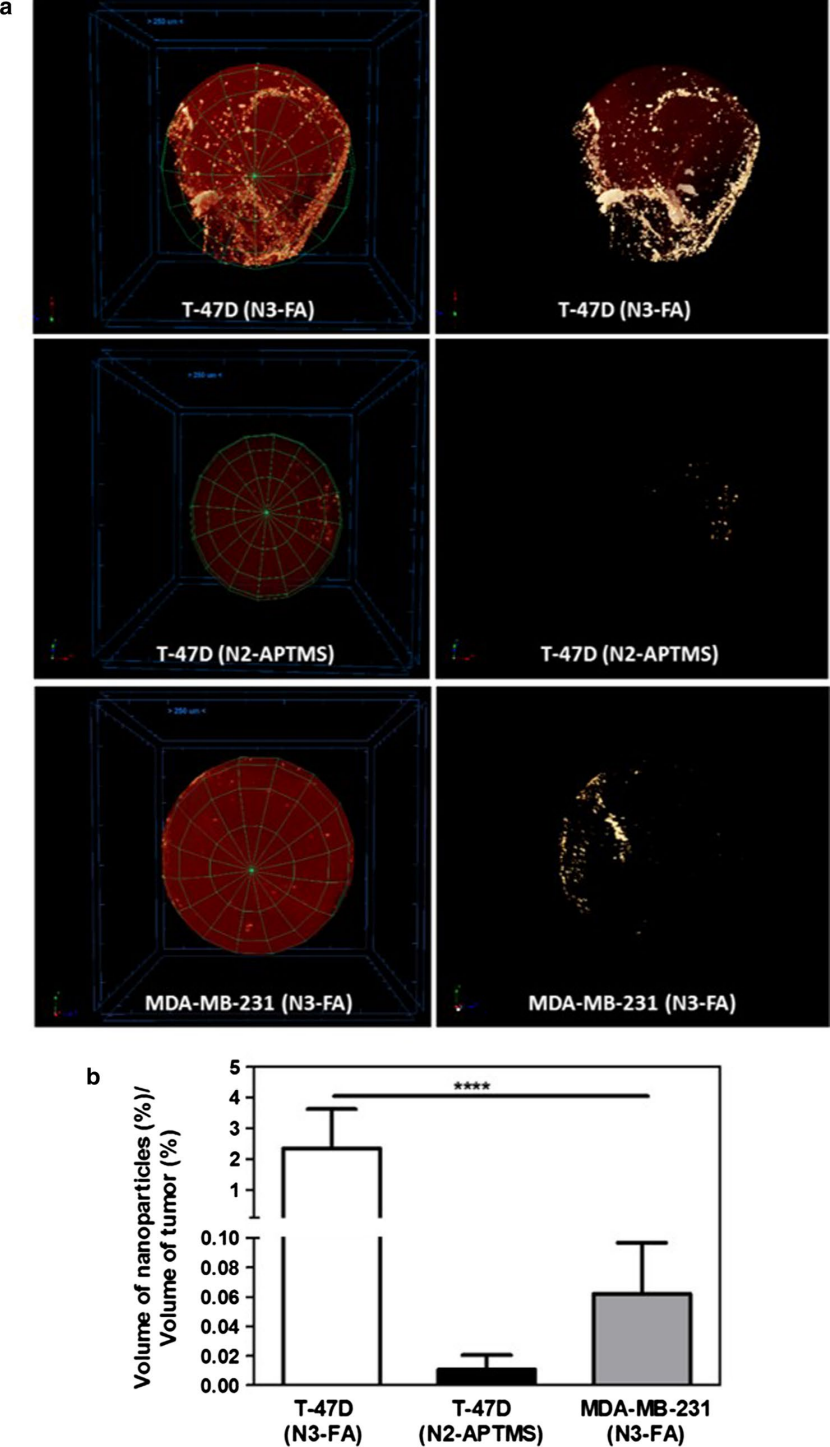
Biodistribution of APTMS conjugated (N2-APTMS), and folic acid-functionalized Gd_2_O_3_:Eu^3+^ nanoparticles (N3-FA) in breast cancer tumors. **a** CT images of different tumors showing biodistribution of N2-APTMS and N3-FA at 2 h after i.v. injection, **b** quantification data representing % volume of tumors occupied by % volume of Gd_2_O_3_:Eu^3+^ nanoparticles. ****P < 0.0001 vs. T-47D tumors with N3-FA treatment using 1-way ANOVA with a Bonferroni post-test

Computed tomography (CT) is one of the most powerful and clinically used techniques to detect tumors. CT possesses the advantage of obtaining unlimited penetration depth that allows the detection of deep lying tumors. Therefore, we hypothesized that a combination of CT and fluorescence imaging using FA-Gd_2_O_3_:Eu^3+^ nanoparticles would enhance the in vivo tumor detection. Mice bearing T-47D or MDA-MB-231 subcutaneous tumors received an i.v. inoculation of either N3-FA or N2-APTMS. Two hours later the mice were euthanized and the tumors were extracted to carry out CT imaging. Intense CT signals due to the presence of Gd_2_O_3_:Eu^3+^ nanoparticles were observed in tumors (Fig. 8a). As expected the signal and the amount of nanoparticles was significantly higher in T-47D tumors when the nanoparticles were functionalized with folic acid (N3-FA) when compared to the signal due to N2-APTMS nanoparticles. In addition the amount of N3-FA nanoparticles was significantly lower in MDA-MB-231 tumors that have low expression of Folr1 when compared to T-47D tumors that are having high expression the receptor (Fig. 8a, b). Together, these results demonstrate the specificity of FA-conjugated Gd_2_O_3_:Eu^3+^ nanoparticles towards Folr^hi^ tumors and their potential as a targeted CT imaging agent for clinical applications.

## Discussion

Breast cancer is the most common cause of cancer-related death among women, and it is estimated that more than 40,000 women in the USA alone will die because of it in 2017 [41]. To increase the chances of successful treatment or survival, it is critical to detect breast cancer as early as possible. Despite the progress in screening with mammography, there is still a significant amount of false-negative results (10–25%) that will lead to therapeutic delay, thus increasing the risk of developing metastasis by the time of detection [42]. Therefore, the development of new diagnostic tools for breast cancer detection is of vital importance. We reported here the development and characterization of folic acid-functionalized luminescent Gd_2_O_3_:Eu^3+^ nanoparticles as a new diagnostic tool for breast cancer detection alone or in combination with CT imaging.

Gd_2_O_3_:Eu^3+^ nanocrystals were produced using sucrose combustion synthesis, and nanoparticles exhibited good crystallinity, narrow size distribution, and optimal quantum yield. Compared to other conventional synthesis methods of Gd_2_O_3_:Eu^3+^ nanoparticles such as sol–gel, hydrothermal and spray pyrolysis, combustion synthesis is a low cost and rapid synthesis procedure to produce nanoparticles with improved photoluminescence properties [43, 44]. The QY of the synthesized nanoparticles strongly depended upon the doping concentration of the activator ion (Eu^3+^). We found 5% Eu^3+^ was optimum to obtain highest QY (~ 41%) and an average decay time of ~ 1 ms. Surface functionalization with folic acid reduced the QY of Gd_2_O_3_:Eu^3+^ nanoparticles to ~ 35% due to increased non-radiative recombination process. Nevertheless, the obtained values indicate that FA-conjugated Gd_2_O_3_:Eu^3+^ nanoparticles (N3-FA) are a good candidate for in vivo bioimaging.

Most of the nanoparticles used for drug delivery or cancer imaging application are 50–150 nm in diameter in order to take advantage of the passive targeting of tumor vasculature via EPR effect [45]. The specificity and efficiency of nanoparticles can be improved by surface modification with a specific targeting ligand. In view of that, the surface of Gd_2_O_3_:Eu^3+^ was functionalized with folic acid to target Folr1, which is frequently over-expressed in breast cancers. The average size of the FA-conjugated nanoparticles (N3-FA) was 55 ± 6 nm, which lies in the size range for passive targeting via EPR effect. The produced nanoparticles can then use both passive and active targeting to reach the cancer cells. Surface charge is important to avoid agglomeration of the particles that are electrostatically stabilized in suspensions. Zeta potential measurements showed that N1-Bare have poor colloidal stability in PBS with a value of + 9.9 ± 3.22 mV, which could be attributed to the lack of surface reactive groups, such as –OH. However, colloidal stability was improved after surface functionalization with APTMS and FA, reaching a zeta potential of + 23.2 ± 1.84 mV for N3-FA, indicating a decreased risk of aggregation. Overall, N3-FA nanoparticles combine a good size for passive targeting to tumors in vivo as well as a limited risk of aggregation, which is an important characteristic for systemic delivery in patients.

We further showed that FA-functionalized Gd_2_O_3_:Eu^3+^ nanoparticles had low cytotoxic effect in vitro at concentrations up to 50 µg/ml. A slight decrease in cell viability was induced quickly after addition of the nanoparticles, even at low concentration (6.25 µg/ml) and kept increasing over time. Since this decrease was quickly induced, it seems likely that the nanoparticles could partly stop cell cycle and proliferation. Additional experiments would be require to study this matter and, at the moment, we cannot discard the possibility of inducing apoptosis if the treatment was prolonged. However, since the primary goal with this nanoparticle is diagnostic, a single injection would be given to patients. The distribution of the signal would then be measured and the nanoparticles would be cleared out of the body hence reducing the risks of continuous exposure and the induction of serious side effects. This is conforted by the fact that in vivo, N3-FA nanoparticles were well tolerated in mice, with doses up to 300 mg/kg, as there was no mortality or change in body weight during the course of the study. In rats, a single injection of gold nanoparticles, eventually led to side effects almost a month after the inoculation, due to their coating [46]. Additional experiments in a different model, with longer time of study would be needed to clear out this possibility. In the meantime, these results suggested a good biocompatibility of N3-FA, encouraging further research for an application as tumor detection markers.

To assess the targeting potential of these nanoparticles Folr^hi^ cancer cells, we used a combination of in vitro and in vivo characterization using fluorescence and CT imaging. In vitro, N3-FA nanoparticles had a faster and significantly higher uptake in Folr^hi^ cancer cells like T-47D compared to Folr^lo^ cancer cells such as MDA-MB-231. Similarly in tumor-bearing mice, systemically delivered N3-FA nanoparticles accumulated preferentially in T-47D tumors when compared to MDA-MB-231 tumors. This distribution was due to the expression of Folr1 on cancer cells and the presence of FA on the nanoparticles since there was significantly less uptake in T-47D tumors when N2-APTMS were injected. Accumulation of N3-FA and N2-APTMS nanoparticles in MDA-MB-231 and T-47D tumors, respectively, is likely due to passive EPR effect. In addition, the presence of fluorescence in the tumors 2 h after the inoculation indicated that there was retention of the nanoparticles since, at this time, it was not possible to detect nanoparticles in blood circulation. The absence of nanoparticles in the blood after 2 h, also indicated a fast clearance of the nanoparticles, which minimizes the risk of toxic response [47]. In normal tissues, fluorescence was detected in the kidneys, probably due to renal clearance of the nanoparticles. However, there were few, or no N3-FA detected in lungs, liver or spleen in tumor-bearing mice, indicating reduced uptake in non-cancerous tissues, which is required for a cancer probe. CT imaging analysis demonstrated that the combination of CT imaging with N3-FA nanoparticles significantly enhanced tumor detection.

## Conclusion

In summary, we have described the development of folic acid-conjugated Gd_2_O_3_:Eu^3+^ nanoparticles with low toxicity that can be used as a fluorescent probe for the detection of Folr1 breast cancer in vivo. Our work provides major evidence that justify future research focused towards the clinical application of FA-conjugated Gd_2_O_3_:Eu^3+^ for detection of breast cancer using optical imaging. In combination with CT scans, it would be possible to achieve high-resolution imaging and detection of deeply located tumors.

## Additional file

**Additional file 1.** Additional figures.
