# Supplementary material for: Functionalized rare earth-doped nanoparticles for breast cancer nanodiagnostic using fluorescence and CT imaging
Source: J Nanobiotechnology. 2018 Mar 22;16:26. doi: 10.1186/s12951-018-0359-9 (PMC5863469; doi:10.1186/s12951-018-0359-9)
Supplement: Supplementary file 1 — Additional file 1. Additional figures. [file 12951_2018_359_MOESM1_ESM.docx]

**Additional information**

**Functionalized rare earth-doped nanoparticles for breast cancer nanodiagnostic using fluorescence and CT imaging**

Akhil Jain^1, 2, 3^, Pierrick G. J. Fournier^1^, Vladimir Mendoza-Lavaniegos^4, 5^, Prakhar Sengar^1^, ^2, 3^, Fernando M. Guerra-Olvera^1^, Enrique Iñiguez^4, 5^, Thomas G. Kretzschmar^4, 5^, Gustavo A. Hirata^3^, Patricia Juárez*^1^

^1^Biomedical Innovation Department, Centro de Investigación Científica y de Educación Superior de Ensenada (CICESE), Carretera Ensenada-Tijuana No. 3918, Zona Playitas, C.P. 22860, Ensenada, B.C. México.

^2^Posgrado en Física de Materiales, Centro de Investigación Científica y de Educación Superior de Ensenada (CICESE), Carretera Ensenada-Tijuana No. 3918, Zona Playitas, C.P. 22860, Ensenada, B.C. México.

^3^Universidad Nacional Autónoma de México (UNAM) - Centro de Nanociencias y Nanotecnología (CNyN), Km. 107 Carretera Tijuana-Ensenada. Ensenada, B.C., México 22860

^4^Departamento de Geología, Centro de Investigación Científica y de Educación Superior de Ensenada (CICESE), Carretera Transpeninsular Ensenada-Tijuana #318, Zona Playitas, C.P. 22860, Ensenada, B.C. México.

^5^Centro Mexicano de Innovación en Energía Geotérmica (CeMIGeo), Rinconada del Pedregal 95, Pedregal Playitas, C.P. 22860, Ensenada, Baja California.

**Corresponding author:** Patricia Juárez. Biomedical Innovation Department, Centro de Investigación Científica y de Educación Superior de Ensenada (CICESE), Carretera Ensenada-Tijuana No. 3918, Zona Playitas, C.P. 22860, Ensenada, B.C. México.

Email: [pjuarez@cicese.edu.mx](mailto:pjuarez@cicese.edu.mx).

Phone: +52 (646) 175-0500 ext. 27212.


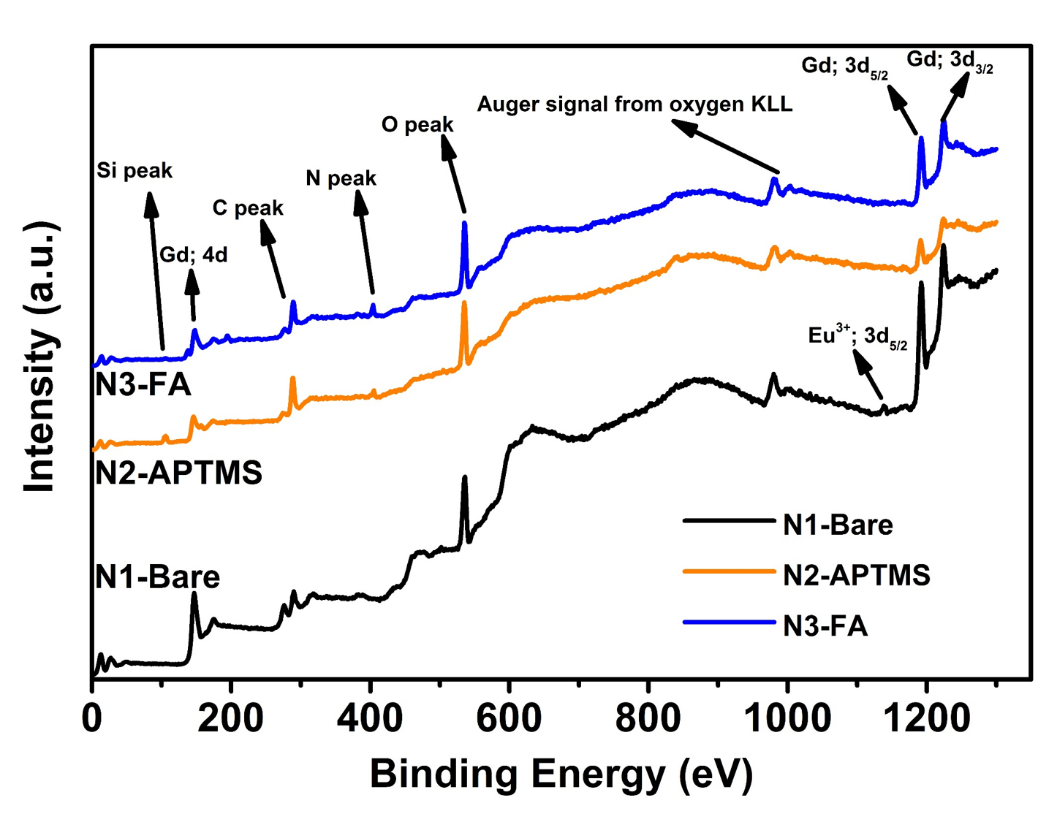


**Figure S1.** Complete XPS spectra of different Gd_2_O_3_:Eu^3+^ nanoparticle system.


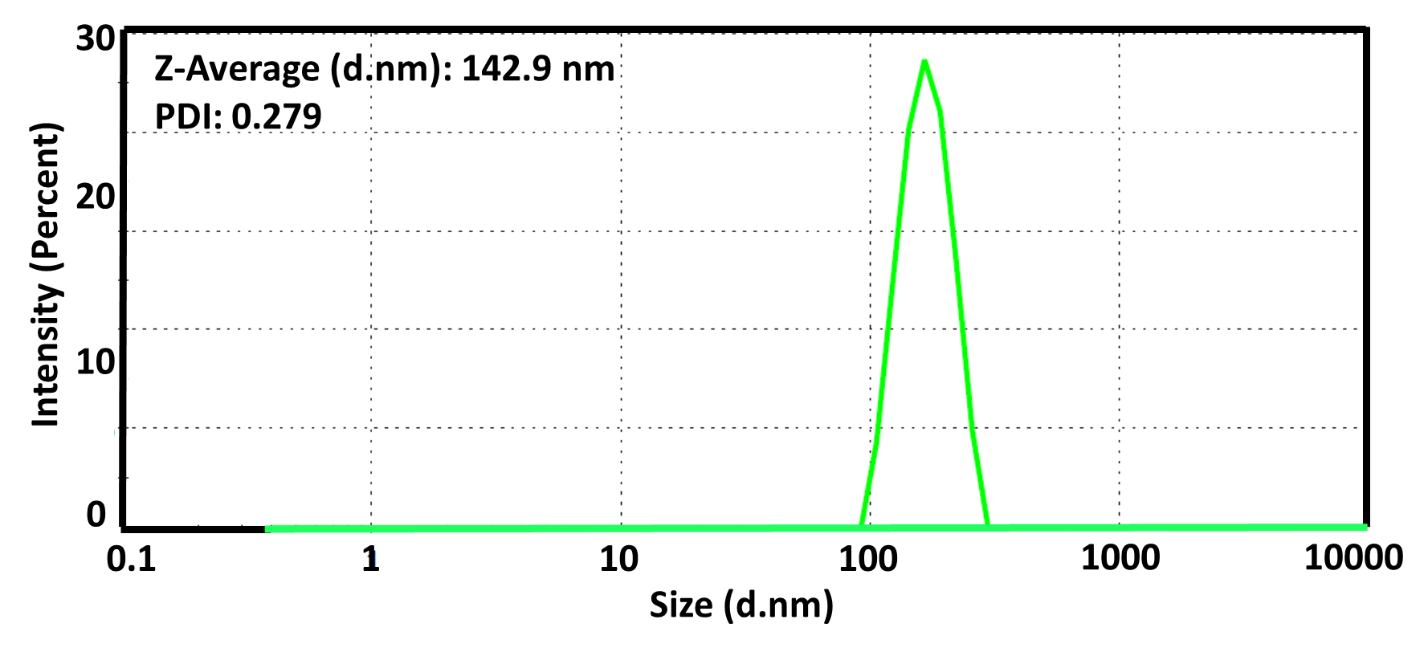


**Figure S2.** Hydrodynamic diameter and polydispersity index (PDI) of folic acid-conjugated Gd_2_O_3_:Eu^3+^ nanoparticles (N3-FA).


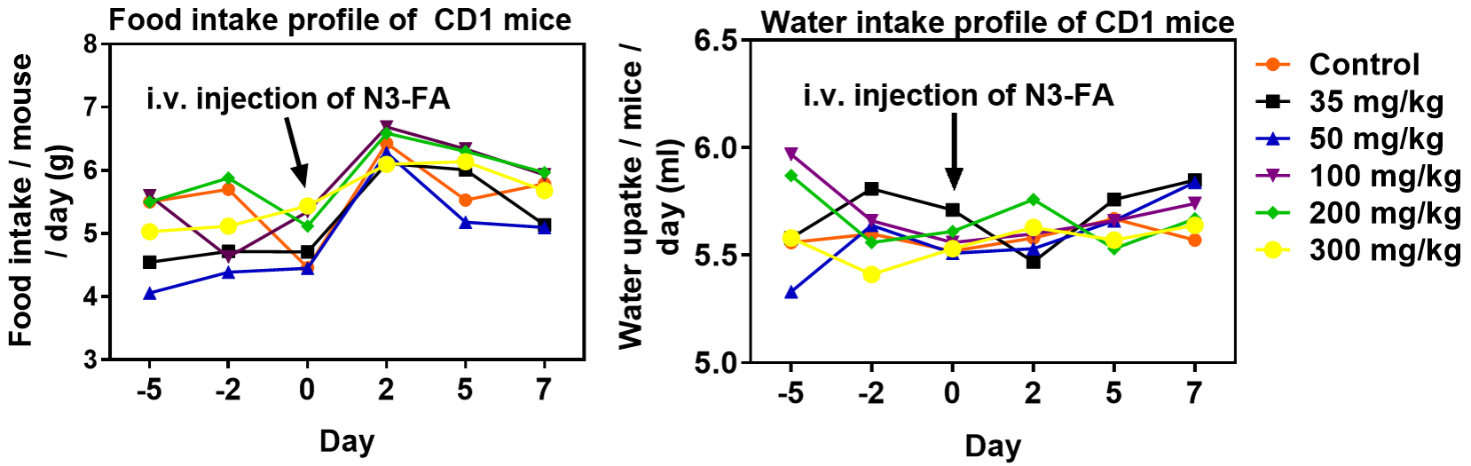


**Figure S3.** Food intake and water uptake of CD1 mice injected with different dose of folic acid-conjugated Gd_2_O_3_:Eu^3+^ nanoparticles (N3-FA).
